# The Brunonian or Molecular Movement

**Published:** 1873-05

**Authors:** James Tyson, S. P. Cutler

**Affiliations:** Memphis, Tenn.


### ARTICLE II.

### *The Brunonian or Molecular Movement.*

By James Tyson, M. D. October No. *Dental Times*, 1872.

REVIEWED BY S. P. CUTLER, M. D., D. D. S., MEMPHIS, TENN.

It seems to me that the above named writer, somewhat misunderstands or misconstrues the sum and substance of my paper in his criticism; that is, if I understand him right.

It is true, I somewhat followed the text of other authors, as quoted in my paper, (and many others not quoted for want of room,) which were consulted at the time, but finding no settled *dictum* on the subject, I simply gave the result of my researches and opinions, written independent of all others.

The above writer can not accuse me of plagiarism, although he may suppose that all I said and described, and ideas advanced, were generally understood by any and all microscopists of any pretensions. But if he will carefully examine my second paper, read on the fifteenth of April, 1872, at the Academy of Sciences, at New Orleans, just one week after the first one was read, and published in the October No. of the *Nashville Medical Journal*, he will see other authors quoted, and the result of further microscopic observations and opinions, somewhat different from those contained in my first paper, read at the same place one week previous, and published in the science column of the *New Orleans Picayune*, and subsequently published in the June No. of the above named journal.

In this second paper (containing the result of an immense number of microscopic observations, of every conceivable substance to be had in the city and elsewhere,) ideas are advanced, that these vibratorial atoms or molecules described, are nothing more or less than the ultimate atoms of matter, with, to myself, good and substantial reason for so thinking, notwithstanding the published opinions of Huxley, Tyndal, Sir Wm. Thompson, and others, that the ultimate atoms of all matter are so small, that they never can be reached.

In this second paper I gave my reasons why these and all other authors on the subject had failed to find them; *i. e.*, they all were hunting for something too small, and used powers too high to see them, which are easily found in everything, by following my plan as given in these papers. I again repeat here, that if any competent microscopist thinks enough about it to follow out my plan as given, he will be certain to see what I have described, and as I saw them, whether he thinks with me or not, that will be a matter of opinion, while the facts are more fully recognized. I also assumed that there is no such thing as dead matter, *so-called*, and that all matter in its atomic form, or condition, possesses life attributes when seen floating in liquids, *i. e.*, motion, life being but a mode of motion, the result of organism, *i. e.*, animal life.

It will be seen by a careful perusal of my first paper, in closing, that I did not regard these forms as organic at all, instead were the component of all kinds of matter, both organic and inorganic. The writer in the *Times* journal, would have us believe, that these minute objects were well understood and recognized by all investigators in microscopy, as though their true place in nature were well understood and recognized. On the contrary, they are imperfectly understood by all investigators up to the present time; which will be readily seen by perusal of the labors of the following investigators, under various heads, besides others not mentioned.

Clifford, Darwin, Helmholtz, Doner, Pouchet, Dr. Grenhow, Beale, Glugey, Cagniard de la Tour, Pflume, Tyndall, Ure, Smith, Sir H. Holland, De Saussuire, Dr. Stanhouse, Beauchamp, Virchow, Leidy, Chatman, Kircher, Pasteur, Nyander, Henle, De la Rive, Schleiden, Bruche, Carpenter, Hillyard, Winlow, Banson, Schwan, Gay Lussac, Reaumer, Eisalt, Dr. Budd, Humboldt, Liebig, Henfrey, Ehrenbergh, Dancer, Cavier.

I find no definite or settled conclusions of their true place in nature, and only passing notices taken of them, and not regarded even as of much importance in the catagory of microscopic science.

Writer says, "So early as 1827, Dr. Robert Brown of Edinburgh, announced, that it was by no means confined to the favilla (speaking of pallin grains,) but was possessed by numerous other substances, organic and inorganic, and therefore in no way characteristic of life." Writer further says, Indeed all substances, when minutely divided, exhibit it."

Did the above writer first publish the above statements as they are substantially stated in my paper referred to? He further says, in the same sentence, "although the different substances which are light, as particles of indigo, or gumboge, requiring to be less minutely divided than those which are specifically heavy, as iron or other metal." This latter statement is substantially an error; showing conclusively to my mind, the want of a proper knowledge of the subject he pretends to be so familiar with. The statement is wholly unfounded in fact; let any one thoroughly versed in microscopic knowledge, follow out my directions given in the paper (so rudely criticized by the writer,) and let him apply the micromitic, and accurately measure the diameters of all the substances given in my paper, and he will find that there is really no practical difference in the average sizes of each substance, the range of sizes being just about the same. In this statement, I challenge all opposition to the contrary, including my opponent in this challenge; none to be so harshly criticised by any one clearly so unfamiliar with the subject, excites pity rather than contempt.

Further on, the writer says, "In almost all instances, however, the degree of division is so small as to acquire a power of at least 200 diameters, and in the case of heavier substances, combination necessary is so great, that 400 or 500 diameters may be required to exhibit it."

In these statements the writer has given the powers as given in my paper, for what he calls the heavier substances, without any quotation marks; he may have already given them in some former passage; if so, I stand convicted, as I had not seen the same; if he gets them from any other writer, he has not given the proper acknowledgements. I wait for information. In his speaking of different powers to see the atoms, I will award to him the credit, as I gave no such distinctions; so far as my observations are concerned, the same power answers equally well for all bodies, there being no recognizable difference, as I have already stated above. From those signal mistakes, or errors on the writer's part,

I am satisfied of his want of accurate knowledge on the subject. Any one who will take the trouble to examine the paper of mine referred to, will towards the end, or latter part, see that I did not regard these the smallest of all objects as organic, but to the contrary, as belonging to all bodies; in this respect the writer has done me injustice, and misrepresented my position.

The directions the writer gives for preparing specimens are substantially those to be found in my paper, he failing to put any quotation marks.

The writer's statement, that these atoms have but the molecular motion and do not change place, is simply a mistake, and any one familiar with their motions will readily discover it, as they do move about more or less, but when in dense masses, of necessity, their movements in this respect are embarrassed to a certain extent. The solitary motion as described by Hillyard, referred to in my paper, is also easily recognized.

I claim that I have found the same phenomenon in everything examined, and that my examinations have been more extended in this direction than any one else's, to my knowledge; I claimed that those same little forms composed the terminus—a broad statement truly, and in my subsequent paper I claimed for them the finality or ultimate of matter, *i. e., ultimate atoms or molecules.*

The writer in the *Times* journal speaks in strong terms, as though he thought I had committed a most signal blunder. The writer calls this motion the Brunonian motion, in honor I suppose of Dr. Robert Brown, of Edinburgh, though none of the above writers named, I believe, mention the fact; if so, it has slipped my memory. Dr. Beale describes the oscillary motion very well in some respects, but does not describe the other motions at all. Dr. T. C. Hillyard recognizes two motions only.

